# Gene expression predictions and networks in natural populations supports the omnigenic theory

**DOI:** 10.1186/s12864-020-06809-2

**Published:** 2020-06-22

**Authors:** Aurélien Chateigner, Marie-Claude Lesage-Descauses, Odile Rogier, Véronique Jorge, Jean-Charles Leplé, Véronique Brunaud, Christine Paysant-Le Roux, Ludivine Soubigou-Taconnat, Marie-Laure Martin-Magniette, Leopoldo Sanchez, Vincent Segura

**Affiliations:** 1 BioForA, INRAE, ONF, Orléans, France; 2 BIOGECO, INRAE, Univ. Bordeaux, Cestas, France; 3 Institute of Plant Sciences Paris-Saclay (IPS2), CNRS, INRAE, Université Paris-Sud, Université d’Evry, Université Paris-Saclay, Gif sur Yvette, France; 4 Institute of Plant Sciences Paris-Saclay (IPS2), CNRS, INRAE, Université Paris-Diderot, Sorbonne Paris-Cité, Gif sur Yvette, France; 5 MIA-Paris, AgroParisTech, INRAE, Paris, France; 6 AGAP, Université Montpellier, CIRAD, INRAE, Montpellier SupAgro, Montpellier, France

**Keywords:** Core, Peripheral, Boruta, Machine learning, *Populus nigra*

## Abstract

**Background:**

Recent literature on the differential role of genes within networks distinguishes core from peripheral genes. If previous works have shown contrasting features between them, whether such categorization matters for phenotype prediction remains to be studied.

**Results:**

We measured 17 phenotypic traits for 241 cloned genotypes from a *Populus nigra* collection, covering growth, phenology, chemical and physical properties. We also sequenced RNA for each genotype and built co-expression networks to define core and peripheral genes. We found that cores were more differentiated between populations than peripherals while being less variable, suggesting that they have been constrained through potentially divergent selection. We also showed that while cores were overrepresented in a subset of genes statistically selected for their capacity to predict the phenotypes (by Boruta algorithm), they did not systematically predict better than peripherals or even random genes.

**Conclusion:**

Our work is the first attempt to assess the importance of co-expression network connectivity in phenotype prediction. While highly connected core genes appear to be important, they do not bear enough information to systematically predict better quantitative traits than other gene sets.

## Background

Gene-to-gene interaction is a pervasive although elusive phenomenon underlying phenotype expression. Genes operate within networks with more or less mediated actions on the phenome. Systems biology approaches are required to grasp the functional topology of these networks and ultimately gain insights into how gene interactions interplay at different biological levels to produce global phenotypes [1]. New sources of information and their subsequent use in the inference of gene networks are populating the wide gap existing between phenotypes and DNA sequences and, therefore, opening the door to systems biology approaches for the development of context-dependent phenotypic predictions. RNA sequencing (RNA-seq) is one of such new sources of information that can be used to infer gene networks [2].

Among the many works on gene network inference based on transcriptomic data, two recent studies aimed at characterizing the different gene roles within co-expression networks [3, 4]. Josephs et al. [3] studied the link between gene expression, gene connectivity [5], divergence [6] and traces of natural selection [7, 8] in a natural population of the plant *Capsella grandiflora*. They showed that both connectivity and local regulatory variation on the genome are important factors, while not being able to disentangle which of them is directly responsible for patterns of selection among genes. Mähler et al. [4] recalled the importance of studying the general features of biological networks in natural populations. With a genome-wide association study (GWAS) on expression data from RNA-seq, they suggested that purifying selection is the main mechanism maintaining functional connectivity of core genes in a network and that this connectivity is inversely related to eQTLs effect size. These two studies start to outline the first elements of a gene network theory based on connectivity, stating that core genes, which are highly connected, are each of high importance, and thus highly constrained by selection. In contrast to these central genes, there are peripheral, less connected genes, never far from a core hub. These peripheral genes are less constrained than core genes and consequently, they harbor larger amounts of variation at population levels.

Furthermore, classic studies of molecular evolution in biological pathways can help us understand the link between gene connectivity and traits. Several articles showed that selection pressure is correlated to the gene position within the pathway, either positively [9–14] or negatively [9, 15–17], depending on the pathway. Jovelin et al. [15] showed that selective constraints are positively correlated to expression level, confirming previous studies [18–20]. Montanucci et al. [21] showed a positive correlation between selective constraints and connectivity, although such a possibility remained contentious in previous works [22, 23].

While Josephs’ [3] and Mähler’s [4] studies framed a general view of genes organization based on topological features described in molecular evolution studies of biological pathways, a point remains quite unclear so far: to what extent core and peripheral genes based on connectivity within a co-expression network are involved in the definition of a phenotype? One way to clarify this would be to study the respective roles of core and peripheral genes, as defined on the basis of their connectivity within a co-expression network, in the prediction of a phenotype. Even if predictions are still one step before validation by in vivo experiments, they already represent a landmark that may not only be correlative but also closer to causation, depending on the modeling strategy.

Present study aims at exploring gene ability to predict traits, with datasets representing core genes and peripheral genes, as defined by a topological based model. By making use of two methods to predict phenotypes of available traits, a classic additive linear model, and a more complex and interactive neural network model, we further aimed at studying the mode of action of each type of genes, in order to gain insight into the genetic architecture of a relatively large range of complex traits. On the one hand, genes that are better predictors with an additive model are supposed to have an overall less redundant, more additive, direct mode of action. On the other hand, genes being better predictors with an interactive model are supposed to operate with high pervasiveness and redundancy, through high connectivity. It is not evident to assign a priori a preferential mode of action and respective roles to core versus peripheral genes. We could assume the former to be downstream genes in biological pathways, closer to the phenotypic expression. The latter could be upstream genes, further away from the phenotype. However, such hypotheses would require levels of data integration that might not be easily available. More readily accessible would be the question of the extent to which connectivity of core genes is captured by models that are sensible to interactivity, involving high but selectively constrained expression levels [15, 21]. With a lower variation, we also expect core genes to be worse predictors for traits than peripheral genes unless the former also bear larger effects.

To answer the questions concerning the respective roles of core and peripheral genes on phenotypic variation, we have sequenced the RNA of 459 samples of black poplar (*Populus nigra*), corresponding to 241 genotypes, from 11 populations representing the natural distribution of the species across Western Europe. We also have, for each of these trees, phenotypic records for 17 traits, covering the growth, phenology, physical and chemical properties of wood. They cover two different environments where the trees were grown in common gardens, in central France and northern Italy. With the transcriptomic data, we built a co-expression network in order to define contrasting gene sets according to their connectivity within the network. We then asked whether these contrasting sets differed in terms of both population and quantitative genetics parameters and quantitative trait prediction.

## Results

### Wood samples, phenotypes, and transcriptomes

**Fig. 1:**
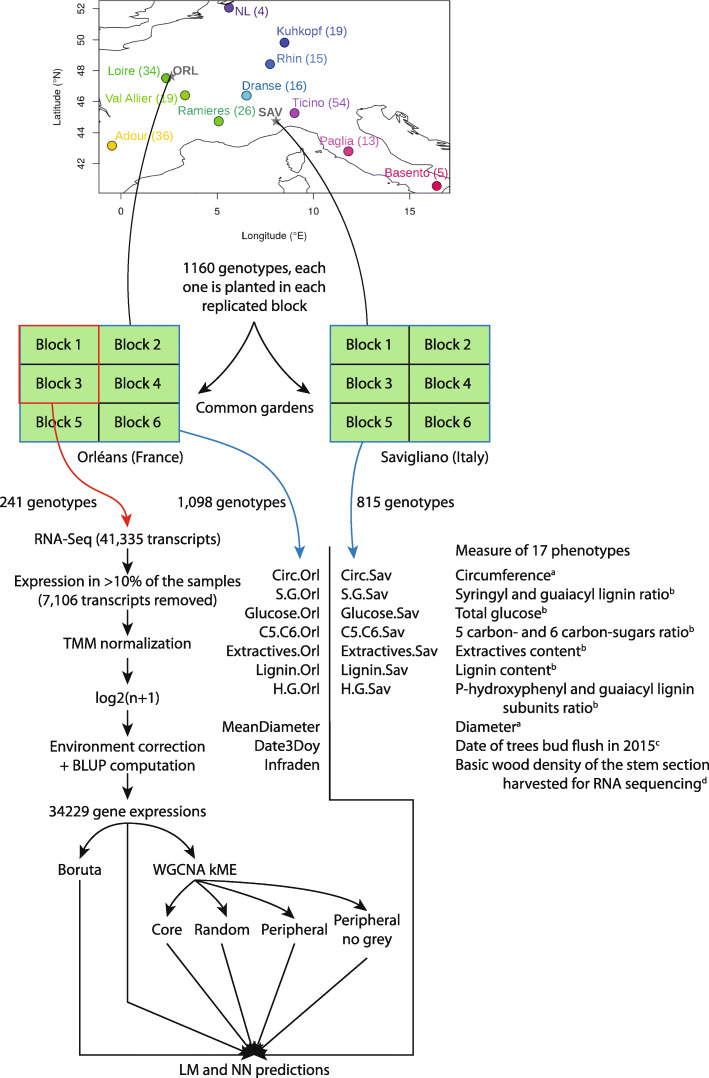
General sketch of the experiment. From the top to the bottom: Map of the location of the different populations sampled for this experiment, the number of individuals used for the RNA sequencing is indicated between parentheses. From these populations, genotypes were collected and planted in 2 locations (Orléans, in central France, and Savigliano, in northern Italy). At each site, we planted 6 clones of each genotype, 1 in each of the 6 blocks, and their position in each block was randomized. For all the blocks, we collected phenotypes: 10 in Orléans (circumference, S/G, glucose, C5/C6, extractives, lignin, H/G, diameter, infradensity and date of bud flush) and 7 in Savigliano (circumference, S/G, glucose, C5/C6, extractives, lignin, H/G). Only on the clones of 2 blocks in Orléans, we performed the RNA sequencing and treatment of data. The treated RNA-seq data were used with different algorithms and in different sets to predict the phenotypes measured on the same trees (in Orléans) or on the same genotype but on different trees (in Savigliano). Trait category: ^*a*^Growth, ^*b*^Chemical, ^*c*^Phenology, ^*d*^Physical

Wood collection and phenotypic data have been previously described [24]. Further details are provided in the “Methods” section. The complete pipeline is sketched in Fig. 1. Briefly, we are focusing on 241 genotypes coming from different natural populations in western Europe and planted in 2 common gardens (to avoid the confounding between genetic and large environmental effects) at two different locations: Orléans (central France) and Savigliano (northern Italy). Each common garden is composed of 6 replicated and randomized complete blocks. A total of 17 phenotypic traits have been collected on these genotypes (7 traits in common between the two locations, 3 unique to Orléans). These traits could be organized into four categories depending on the biological process they described (Fig. 1), and they appeared to be quite diverse in terms of genetic control with marker-based heritability estimates ranging between 0.05 and 1 (data not shown). In Orléans only, we used 2 clonal trees per genotype (from 2 blocks) to sample xylem and cambium during the 2015 growing season, and pooled them for RNA sequencing. No tree from Savigliano was used for RNA-seq. Because of sampling and experimental mistakes that were further revealed by the polymorphisms in the RNA sequences, we ended up with 459 samples for which we confirmed the genotype identity (comparison to previously available genotyping data from an SNP chip [25]. These samples corresponded to 218 genotypes with two biological replicates and 23 genotypes with a single biological replicate.

We mapped the sequencing reads on the *Populus trichocarpa* transcriptome (v3.0) to obtain gene expression data. We removed from the data the transcripts for which we did not have at least one count in 10% of the individuals, yielding 34,229 transcripts. We then normalized the data (with TMM) and stabilized the variance (with *log*_2_(*n*+1)). RNA collection lasted over a 2-weeks period, with varying weather conditions along the days. We did PCA analyses on the cofactors that were presumably involved in the experience, to look whether any confounding effect could be identified (Suppl. Fig. 1). No clear segregation was found for any of those, except for the ones associated with block, date and hour of sampling. We used a linear mixed-model framework to correct the effects of these cofactors on each transcript (see the “Methods” section for a formal description of the model used), with R (v3.6.3) [26] and the breedR R package (v0.12.2) [27], and further computed from the models the complete BLUP for each genotype. Hereafter, we refer to this set of BLUPs for the 34,229 transcripts as the full gene set (83% of annotated transcripts).

### Clustering and network construction

**Fig. 2:**
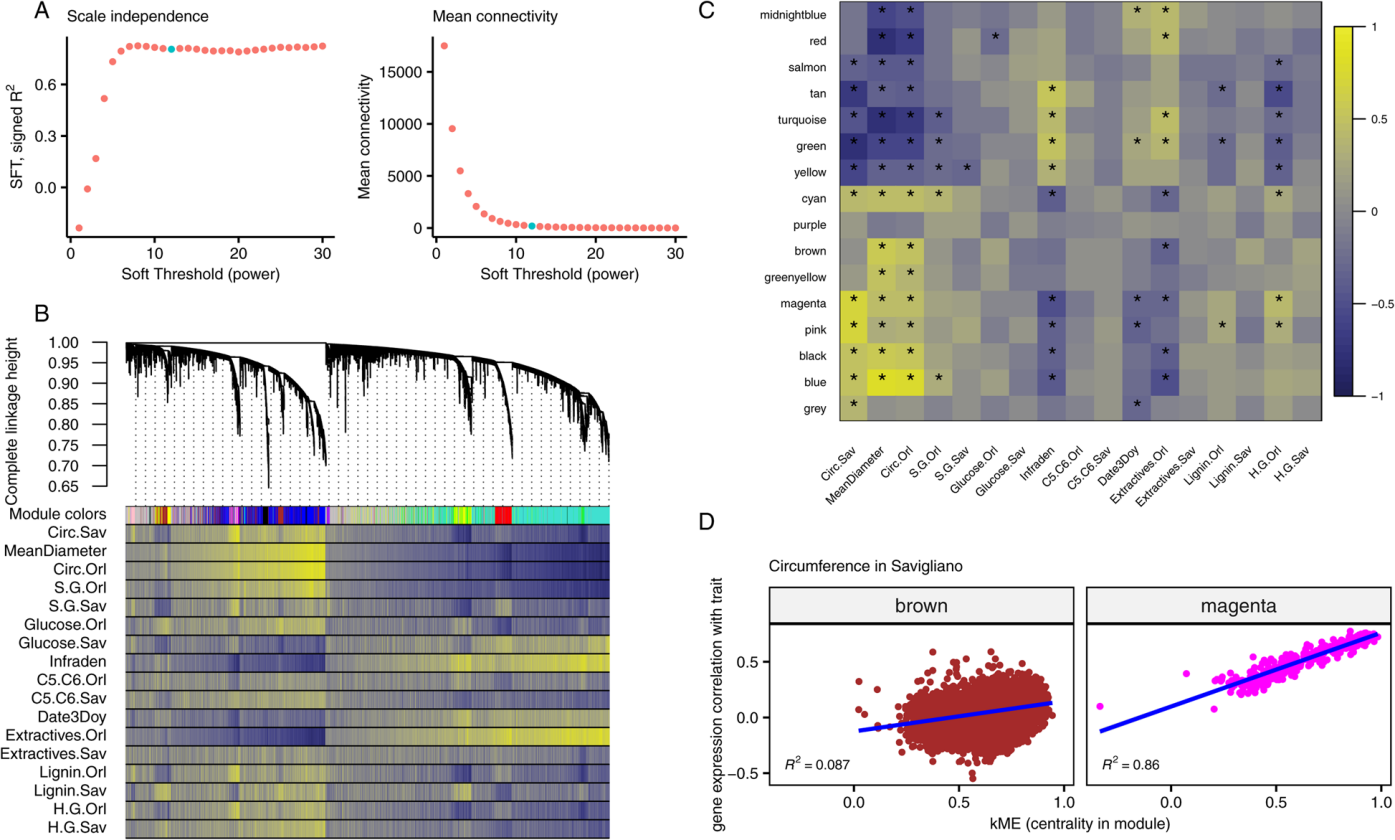
WGCNA analysis of gene expression data. **a**: Selection of the soft threshold (green dot) based on the correlation maximization with scale-free topology (left panel) producing low mean connectivity (right panel). **b**: Gene expression hierarchical clustering dendrogram, based on the Spearman correlations (top panel), resulting in clusters identified by colors (first line of the bottom panel). Spearman correlations between gene expressions and traits values are represented as color bands on the other lines of the bottom panel, from highly negative correlations (dark blue) to highly positive correlations (light yellow), according to the scale displayed in panel C. **c**: Spearman correlation between eigengenes (the best theoretical representative of a gene expression module) of modules identified in the previous panel and traits, again on a dark blue (highly negative) to light yellow (highly positive) scale. Stars in the tiles designate correlations with a significant *p*-value (lower than 5%) after Bonferroni correction. D: Focus on two modules from the previous graph, representing gene expression correlation with the circumference in Savigliano against centrality in the module. These two panels represent the strongest (right panel, magenta module, *R*^2^ = 0.86) and the weakest (left panel, brown module, *R*^2^ = 0.09) correlations with the corresponding trait

The commonly used approach to build a signed scale-free gene expression network is to use the weighted correlation network analysis (implemented in the WGCNA R package (v1.68) [5]), using a power function on correlations between gene expressions. We chose to use Spearman’s rank correlation to avoid any assumption on the linearity of relationships. The scale-free topology fitting index (*R*^2^) did not reach the soft-threshold of 0.85, so we chose the recommended power value of 12, corresponding to the first decrease in the slope growth of the index, resulting in an average connectivity of 195.2 (Fig. 2a). We detected 16 gene expression modules (Suppl. Table 1) with automatic detection (merging threshold: 0.25, minimum module size: 30, Fig. 2b). Spearman correlations between phenotypic and expression data, presented in the lower panel of Fig. 2b below the module membership of each gene, displayed a structure when the order followed the gene expression tree. The traits themselves were line ordered according to clustering on their scaled values to represent their relationships (Suppl. Fig. 2). Interestingly, most patterns in the correlation between expression and traits did not follow what we would have expected, a certain similarity between sites for a given trait (5 traits with unexpected behavior out of 7 with data in both geographical sites: Circ, S.G., Glucose, Lignin and H.G.). For instance, in the group composed of S/G ratios and glucose composition, the patterns were more similar for different traits in the same site than for the same trait in the different sites (Fig. 2b). Complex shared regulations mediated by the environment seem to be in control of these phenotypes, suggesting site-specific genetic control. Otherwise, glucose composition in Savigliano, wood basic density, and extractives in Orléans presented similar patterns, contrarily to what would be expected from the low phenotypic correlations observed between these traits. These results from the comparative analysis of correlations pinpoint some underlying links between traits that are not obvious from factual phenotypic and genetic correlations between traits.

To get further insight into the relationships between module composition and traits, we looked at the strongest correlations (positive and negative) between the best theoretical representative of a gene expression module (eigengene) and each trait, in order to identify genes in relevant modules with an influence on the trait (Fig. 2c). Following a Bonferroni correction of the *p*-values provided by WGCNA, only 80 correlations remained significant (*p*≤0.05) out of the initial 272 traits by module combinations. Six traits displayed no significant correlations with any module (Glucose.Sav, both C5.C6, Extractives.Sav, Lignin.Sav and H.G.Sav) and 1 module was not significantly correlated with any of the traits studied (purple, Fig. 2c). For those modules showing significant correlations with traits, it was also observed a significant correlation between those expression versus trait correlations and the centrality in the modules (represented by the kME, the correlation with the module eigengene). Conversely, no correlation was found in poorly correlated modules (Fig. 2d, Suppl. Fig. 3). In other words, there was a three-way correlation. The genes with the highest kME in a given module were the most correlated to the eigengene and, consequently, were also the most correlated to those traits with the largest correlation with the module eigengene. Although this is somehow expected, it underlines the usefulness of kME as a centrality score to further characterize the genes within each module. We thus used this centrality score to define further the topological position of our gene expressions in the network and to serve as a basis for role comparisons between genes. For each gene, we used its highest absolute score, which corresponds to its score within the module to which it was assigned. We selected the 10% of genes with the highest global absolute scores to define the core genes group, and 10% with the lowest global absolute scores to define the peripheral genes group. Finally, we selected 100 samples of 3422 (10%) random genes as control groups (Suppl. Fig. 4, bottom panel).

One particular module from the WGCNA clustering is the grey module. This module gathers genes with low membership. In our case, it is the 2nd largest module, with 7674 genes (23% of the full set). It gathers the vast majority of genes with very low kME (Suppl. Fig. 4, bottom panel) and 99% of the peripheral genes set (Suppl. Table 2). While it is typically discarded in classic clustering studies, we chose to maintain it and rather understand its composition and role. Therefore, the peripheral gene set gathering the 10% lowest kME grey module genes was added to the comparative study. An extra gene set was considered to complete the set of gene scenarios, one that involved low kME genes that did not belong to the grey module (subsequently called "peripheral NG", NG for "no grey").

### Heritability and population differentiation of modules

**Fig. 3:**
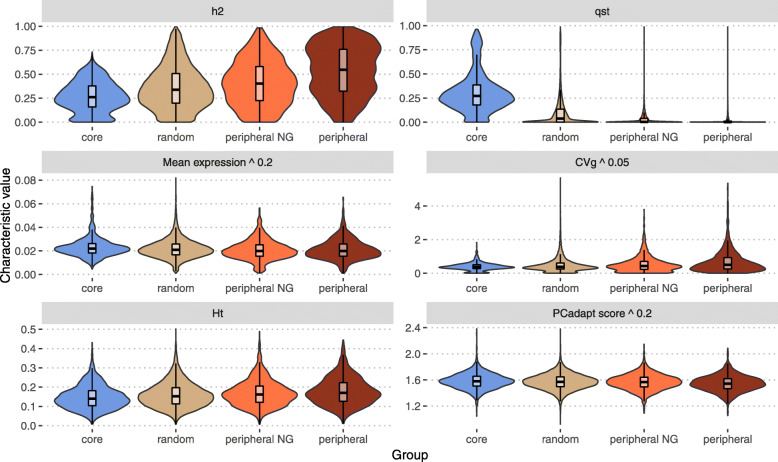
Characteristics of several gene sets. Heritability *h*^2^, differentiation *Q*_*ST*_, gene mean expression (in counts per million, power 0.2), genetic variation coefficient *CV*_*g*_ (power 0.05), overall gene diversity *Ht* and *PCadaptscore* (power 0.2) violin and box plots with median (black line) and interquartile range (black box) for each of the core (in blue), random (in grey), peripheral NG (in orange) and peripheral (in brown) gene sets

To get further insights into the biological role of core and peripheral genes at population levels, we compared the distribution of various characteristics among gene sets (Fig. 3): gene expression level, several classical population statistics, including heritability (*h*^2^), coefficient of quantitative genetic differentiation (*Q*_*ST*_), coefficient of genetic variation (*CV*_*g*_), gene diversity (*Ht*), and a contemporaneous equivalent to *F*_*ST*_ for genome scans (*PCadaptscore*). Gene expression level, *h*^2^, *Q*_*ST*_, and *CV*_*g*_ were computed from gene expression data, while *Ht* and *PCadaptscore* [28] were computed from polymorphism data (SNP) and averaged per gene model. For more details see the “Methods” section.

Globally, there is a clear trend from core to random, to peripheral NG and to peripheral among these characteristics: with an increase for *h*^2^, *CV*_*g*_ and *Ht*, and a decrease for *Q*_*ST*_, expression and *PCadaptscore*. The only differences that are not significant according to a Wilcoxon rank sum test and after Bonferroni correction are those between peripheral NG and peripheral sets in gene expression (*p*-value = 0.14) and between random and peripheral NG sets in the *PCadaptscore* (*p*-value = 0.39). All the other comparisons have *p*-values below 0.001.

Altogether, these statistics showed clear differences between core and peripheral genes: core genes are highly expressed, highly differentiated between populations in their expression and by their allele frequencies at linked markers, and with generally low levels of genetic variation. Contrastingly, peripheral genes are poorly expressed, poorly differentiated between populations, with generally higher genetic variation.

### Boruta gene expression selection

In addition to previous gene sets building (full, core, random, peripheral NG and peripheral), we wanted to have a set of genes being relevant for their predictability of the phenotype. Our hypothesis here was that this set would be the one that enables the best prediction of a given trait but with a limited gene number. For that purpose, we performed a Boruta (Boruta R package (v6.0.0) [29]) analysis on the full gene set with 60% of the genotypes (training set). This algorithm performs several random forests to analyze which gene expression profile is important to predict a phenotype. We tested 4 different threshold *p*-values for this algorithm, as we originally wanted to relax the selection and eventually get sets of different sizes. However, the number of genes selected decreased while relaxing the *p*-value (613, 593, 578 and 578 respectively for 0.01, 0.05, 0.1 and 0.2). Among the 4 *p*-values tested, 190 genes were systematically selected (114 are core, 2 are peripheral NG and 2 are peripheral genes), and 153 were selected on 3 of the 4 *p*-value sets (73 are core, 4 are peripheral NG and 4 are peripheral genes). By averaging across the 4 *p*-values tested, there was a 6.61 mean over-representation of core genes and 0.30 and 0.31 under-representation of respectively peripheral NG and peripheral genes (Suppl. Fig. 5). In the end, with a *p*-value of 0.01, a pool of 613 unique gene expressions was found to be important to predict our phenotypes. Traits with the highest number of important genes are related to growth. For the other traits, we always have more genes selected when the trait is measured in Orléans compared to Savigliano (respective medians of 23 and 10), which fits well with the fact that RNA collection was performed on trees in Orléans. On average, genes that were specific to single traits represented 94% of selected genes, 1 gene was shared across sites for a given trait, genes shared by trait category (growth, phenology, physical, chemical) were 4%, and genes shared among all traits were 2%.

### Phenotype prediction with gene expression

**Fig. 4:**
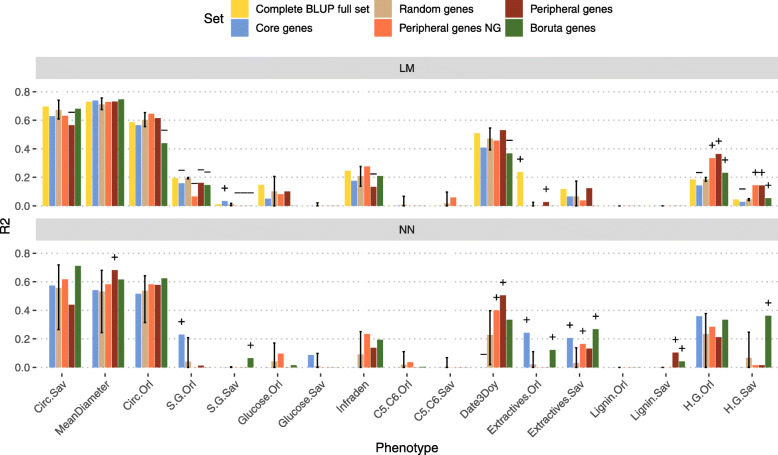
Predictions scores on test sets. Predictions scores on test sets (*R*^2^ on the y axis) for the 2 algorithms (LM Ridge, top panel; neural network, bottom panel) for each phenotypic trait (on the x axis). The color of each bar represents the gene set that has been used for the prediction. Intervals for the random set represent the 95% confidence interval of the distribution of the 100 different realizations, while the height of the bar corresponds to the median. The "+" and "-" signs above the bars indicate predictions respectively above and below the 95% confidence interval of the random set

For our 6 genes sets (full, core, random, peripheral NG, peripheral and Boruta), we trained two contrasting classes of models to predict the phenotypes: an additive linear model (ridge regression, LM) and an interactive neural network model (NN). For the former, we used ridge regression to deal with the fact that for all gene sets the number of predictors was larger than the number of observations. For the latter, we chose NN as a machine-learning method, which is not subjected to dimensionality problems [30] and is able to capture interactions without a priori explicit declaration between the entries, here gene expressions. These contrasting models let us capture more efficiently either additivity or interactivity and are thus likely to inform us about the preferential mode of action of each gene set depending on their relative performances in predictability. Figure 4 shows that for LM with ridge regression, the best gene set to predict phenotypes was on average the full set, as expected because it contains more information, followed, more surprisingly, by the peripheral and peripheral NG genes set, then the random, core and Boruta sets (respective mean prediction *R*^2^ across all traits of 0.22, 0.21, 0.20, 0.19, 0.18 and 0.17). However, these advantages among sets were relatively small, when compared to the framework of random sets given by the 95% confidence interval from 100 realizations (95% CI, Fig. 4). Specifically, no differences were observed between random and alternative gene sets for most of the traits, with no overall set outperforming clearly the others when accounted only for traits showing significant differences with respect to 95% CI (Suppl. Fig. 6). For NN and on average terms of *R*^2^, random genes were the worst set, followed by core, peripheral, peripheral NG and Boruta sets (respective mean prediction *R*^2^ across all traits of 0.14, 0.16, 0.17, 0.18 and 0.22). Again, advantages were small when compared to the reference 95% CI from random realizations. Unlike LM, however, NN yielded some net advantage for alternative sets with significant traits being mostly upwardly placed in their performances (higher *R*^2^) with respect to the 95% CI. Among the sets with most significant cases were the Boruta, then peripheral genes, peripheral NG genes, and Core genes. We have not been able to compute NN models with the full set as the number of predictors remains too large to be fitted with the computational power being available on computing clusters. Across phenotypes, predictions were generally slightly less variable under NN than under the ridge regression counterpart (interquartile range mean division by 1.12).

To further investigate the behavior of genes with different positions in the network with respect to the prediction model used, we computed 2 types of differences: (i) between LM and NN prediction scores for each gene set, and (ii) between core and peripheral genes sets for LM and for NN models (Suppl. Fig. 7). As a null reference for inference in the comparison between peripheral and core sets, we computed the differences between all the 100 random sets, for a total of 4950 differences corresponding to all pairwise differences, excluding reciprocals and self-comparisons. In the top panel, a positive difference indicates that LM predicted better than NN and *vice versa*, while in the bottom panel, a positive difference indicates an advantage of core genes sets over peripherals and, conversely, a negative difference indicates an advantage of peripheral genes. In any of the two panels, we did not detect any systematic difference, favouring one of the modeling options across traits or one of the gene sets across traits. Moreover, the few cases where a difference is to be noted were certainly due to very poor prediction scores. The only difference that can be noted by its magnitude is the difference between core and both peripheral genes in NN for the date of bud burst (Date3Doy), in favor of the peripheral genes.

Finally, we investigated to what extent trait *h*^2^ or *Q*_*ST*_ would influence the prediction scores of each combination of set and algorithm. We found a positive and significant relationship between trait *h*^2^ or *Q*_*ST*_ and prediction accuracy irrespectively of the gene set or the prediction method considered (Spearman’s rank correlations ranged from 0.36 to 0.56 and from 0.27 to 0.47 for trait *h*^2^ and *Q*_*ST*_, respectively). When looking specifically at sets and methods, we did not find many cases showing significantly higher correlations between *h*^2^ or *Q*_*ST*_ and prediction than the random sets. For *h*^2^, only the Boruta set in LM was above the 95% CI, while for *Q*_*ST*_, only the Complete set in LM and the Boruta and Peripheral sets in NN were above the 95% CI. We further separated traits according to whether their *Q*_*ST*_ was above or below the 99^*th*^ percentile of the *F*_*ST*_. The rationale under this split is that because core genes are more differentiated between populations than random or peripheral genes, we should expect them to predict better those traits with a similar structuration behavior and *vice versa*. We found that traits above the 99^*th*^ percentile of the *F*_*ST*_ were systematically better predicted than less differentiated traits. However, we did not find significant differences between gene groups once the difference between traits was taken into account.

## Discussion

Characterizing the way genes contribute to phenotypic variation could prove highly valuable to better understand the genetic architecture of complex traits. With the advent of omics data, a huge amount of information is nowadays becoming available to fill the gap between variations at the DNA and phenotype levels [31]. Such gap-filling can be obtained from multiple sources. For instance, accounting for the number of shared neighbors between two genes informs on subsequent protein-protein interactions bringing further biological meaning [32]. It is by the use of gene expression data that the present study aimed at gaining insights into the genetic architecture behind complex traits.

One key premise in the study was the availability of a common garden experiment comprising relevant samples of natural variation, in our case black poplar from Western Europe. Such an experimental setting makes it possible to accurately evaluate phenotypes to calibrate and serve as a target for predictions. Indeed, evaluating all the genotypes in a given location with experimental design and replicates enabled to unravel the confounding between genotype and macro-environment (or micro-environment) that typically occur when considering genotypes in the wild [33]. Likewise, RNA-seq data were collected on up to two biological replicates in the common garden and also corrected for environmental and design covariables, to obtain the genotypic BLUP, which is the genetic value of the genotype. Such adjustments at both phenotypic and genomic ends provided proper grounds with reasonable confidence in the absence of undesirable effects for the study of associations between the two sources of data.

Two recent works used RNA-seq in natural populations of plants to build co-expression networks and study the relationship between network topology and patterns of natural selection [3, 4]. While they found differences in natural selection among genes given their connectivity within networks, they did not investigate how these differences affect phenotypic variation. We thus embraced the commonly used WGCNA approach [5] to build the co-expression network within our dataset in order to study the relationship between gene connectivity and phenotypic prediction. This clustering of genes gave us different groups that we found to be differently correlated to traits values and according to sites. However, this method was simply for us a way to obtain a centrality or connectivity score for each gene, with the subsequent possibility to classify them into core and peripherals. The biological interpretation of correlations between gene groups and traits would clearly deserve further work which is beyond the scope of the present study. We based our definition of core and peripheral on Mähler et al. [4], as respectively the 10% most central and most peripheral genes. The only specificity of our work here is that we did not discard, as it is classically done (called pruning in the WGCNA manual), the genes from the grey group, i.e. those showing a poor membership to any other module. We considered instead two alternative peripheral sets by keeping or excluding genes from the grey group. The pertinence of kME as a classification criterion became evident in our study when looking at the differences between core and peripherals in terms of classic quantitative and population genetic parameters. Core genes (high kME) showed high levels of population differentiation, mostly in quantitative genetic terms (*Q*_*ST*_), while being simultaneously less variable than the rest of the genes. Such results would suggest that core genes are genes potentially subjected to divergent selection, with subsequently reduced levels of genetic variation, and involved in local adaptations. Contrarily, peripherals (low kME) showed larger levels of variation with respect to their expression level and little structure across populations, suggesting less selection pressure or weaker connection to selected traits, with mostly stabilizing selection patterns across populations. Therefore, despite the fact that a subdivision in core and peripherals is somehow an oversimplification, an extreme contrast of an otherwise continuous phenomenon, it helped to reveal the different natures of genes characterized by extreme values of kME.

To further test whether this gene categorization matters for trait prediction, we decided to go one step further by trying to predict traits from the different gene sets. We also wanted to have a gene set designed to be composed of good predictors of the traits. We thus used the Boruta algorithm [29] that performs random forest predictions by selecting the genes with the highest prediction importances. We have to keep in mind that random forest algorithms allow for implicit interactions between predictors (here gene expressions [34–36]). Results pinpointed again one feature differentiating the behavior of core and peripheral genes. Cores were largely overrepresented in the different Boruta selections (by at least 38% of Boruta genes), involving systematically the same 114 genes across all threshold *p*-values (or 153 over 3 values). Peripherals were systematically underrepresented to a very large extent (less than 7%). Although the remaining genes, neither cores nor peripherals according to our previous definition, were the majority (53%) among the ones selected by Boruta, they were sampled from a vaster pool of more than 27,000 genes. Another important result from the Boruta selections is the fact that relaxing the *p*-value threshold (from 0.01 to 0.2) did not increase the size of the resulting selection set, while the set itself could change partially in composition across different thresholds. One can assume that relaxing the threshold would lead to increasing the number of features if these acted independently and contributed with novel information. The fact that numbers did not change substantially, while the composition was indeed impacted, leads to thinking that features are deeply interconnected and do not add up independently. This would suggest that different arrangements of genes could contain comparable levels of information or, in other words, that genes bear some redundancy through networks of interactivity.

With these 6 genes sets, we predicted 17 phenotypic traits with 2 alternative algorithms, one expected to capture mostly additivity between predictors (LM), the other one interactivity (NN). As expected, the full set resulted in best predictions with the LM model (NN not available), as it comprised all available genetic information but it rarely predicted above the random 95% CI. Furthermore, core genes were far from being the best set to predict the different traits under either of the two algorithms. Such results would be a priori surprising considering previous statements on the composition of Boruta selection where cores had an important contribution. The key difference, however, is that cores were not the only contributors to the Boruta sets. It seems that cores are able to summarize key information for quality predictions but require a complementary contribution from other interacting genes to round up the optimal set. This is better reflected by the performance of the Boruta set, which obtained the best performance predicting traits under the NN algorithm. To some extent, the NN algorithm exploits the interactivity between features (genes) already present in the Boruta set, itself obtained through the random forest heuristics that are particularly sensitive to interactions. The high connectivity of high kME value core genes is well captured by interaction sensitive algorithms to improve prediction.

In a contrasting way, the core set performed poorly under LM modeling, where the two classes of peripherals obtained the best predictabilities. Such a performance from peripherals is somehow surprising, in the sense that this class of genes, notably the grey module, is usually pruned from transcriptomic studies, while they seem nonetheless to harbor important biological information that is relevant to the trait variation. Judging from the nature of the LM modeling, peripherals would have more a type of additive gene action, which could be in turn a penalizing feature when a reduction in the number of genes operates to focus only on the most relevant ones (*i.e.* underrepresentation of peripherals in Boruta set). Thus, peripherals appear to be relevant when allowed to contribute cumulatively to prediction, although they can be otherwise easily summarized by more integrative genes when variable selection procedures operate to obtain optimal sets. It is important to note, however, that adding peripherals (following an increasing kME) beyond the numbers present in their original sets did not improve predictability (Suppl. Fig. 8), suggesting the existence of a plateau in their capacity to explain trait variation. The low connectivity of peripheral genes, reflecting independent features, is best exploited by linear model approaches capturing mostly additive genetic actions.

Finally, random sets offered a convenient framework for inferences when comparing gene sets. Their performance in terms of predicting quality was never the best under either of the alternative modeling approaches (LM or NN) but was good enough to suggest that relevant information can be nevertheless obtained from many different gene sets, pointing at some degree of pervasive redundancy in the genetic architecture of traits. In practical terms, when a trait prediction is required but there is no biological a priori on the choice of genes, a random set modeled through LM appears like a satisfying solution. This is not far from the SNP based counterpart in genome-wide evaluation [37], where markers are often a choice that is not driven by biological context. However, if some previous selection of genes is required, the combination of Boruta selection and subsequent NN modeling has been shown here to be a good option for predictability on a reduced genic panel. Indeed, Boruta or any other NN option are advantageous alternatives in genomic evaluation for breeding to more classic methods, often based on the imposition of a priori constraints for shrinkage or variable selection [38].

One of the particularities of core genes, that of showing highly structured genetic variation among populations, led us to think that they might be preferentially involved in traits also showing high levels of *Q*_*ST*_. Such a hypothesis was not confirmed by our results, where highly structured traits were generally better predicted than traits with no apparent structure, but with no clear differences in such an advantage between gene sets. Therefore, the highly structured core genes did not contribute to improving the prediction of highly structured traits, suggesting that trait covariation between populations is affected by other genic sources not conveniently unraveled here. It is important to note that prediction quality is highly variable between traits, somehow masking the differences that might be found between gene sets. We have already pinpointed the relevance of kME in establishing a gradient of genes whose extremes show different behaviors in quantitative and population genetics statistics. These extremes also contribute differently to the explanation of phenotypic variability, through the light of different prediction models. One aspect that remained unanswered, however, is to what extent kME is also relevant to prediction without circumscribing our scope to the extremes. When computing the correlations between connectivity (kME) and prediction coefficients (importance in terms of effect) from LM across all the full set of genes, results showed that there are some strong positive correlations for three of the traits (Circ.Orl, S.G.Orl and Extractives.Orl). However, there is not a systematic trend across all the traits, suggesting that other differences in their genetic variability and genomic architectures might be also of importance here.

In the end, differential connectivity as reflected by our kME gradient from gene expressions pinpoints the importance of mechanisms of gene interactions in the genetic architecture of traits. On top of the DNA sequence, the superposing layer of transcriptomics adds up the intermediate pattern of gene interactions and physiological epistasis, before the final level of phenotypic expression [39]. It is important to note, however, that such gene interaction at the transcriptomic level is not directly or necessarily related to epistasis in the context of statistical genetics literature, i.e. the interaction effect between alleles from different loci on a given phenotype [40]. The extent to which connectivity or transcriptomic interactivity relates to that level of epistasis is beyond the scope of current work but clearly deserves further investigation.

## Conclusion

This work shows that all genes seem important to some extent to predict phenotypes. If the Boruta selection leads us to think that core genes may be very important, prediction results across a range of phenotypes underlined that they are not the only ones. The information that those core genes contain has to be completed by other complementary genes. Likewise, on the other extreme of our networks, peripherals seem also to bear enough biological information to build up sounding predictions. Our analytical approach, by looking at the specific roles of genes with different networking connectivities, highlights the importance of the gene system as a whole in explaining phenotypic variation rather than that of particular sets of genes. Our work is globally in accordance with the recent work on the omnigenic model [41, 42], stating that all genes expressed in an organ participate in the traits of that organ. We were also able to predict phenotypes of an organ or at the organism level, with gene expression from another organ. However predicting and explaining are 2 different things, and the information beared by some genes may be too redundant to lead us to good mechanistic models, without further integration of biological information filling the gap between sequences and phenotypes.

## Methods

### Samples collection

As described in previous works [24, 43], we established in 2008 a partially replicated experiment with 1160 cloned genotypes, in two contrasting sites in central France (Orléans, ORL) and northern Italy (Savigliano, SAV). At ORL, the total number of genotypes was 1,098 while at SAV there were 815 genotypes. In both sites, the genotypes were replicated 6 times in a randomized complete block design. The experiments were carried out in accordance with local legislation. At SAV, the trees were pruned at the base after one year of growth (winter 2008–2009) to remove a potential cutting effect and were subsequently evaluated for their growth and wood properties during winter 2010–2011. At ORL, the trees had the same pruning treatment after two years of growth (winter 2009–2010) and were also subsequently evaluated for growth and wood properties after two years (winter 2011–2012). After evaluation, we pruned again for a new growth cycle. In their fourth year of growth of this third cycle (2015), 241 genotypes present in two blocks of the French site were selected to perform sampling for RNA sequencing. In the end, we obtained transcriptomic data from 459 samples, 218 genotypes duplicated in the two blocks and 23 genotypes available from only one block. These 241 genotypes were representative of the natural west European range of P. nigra through 11 river catchments in 4 countries (Fig. 1). More details on the origin of these genotypes including their depositary are available in the GnpISInformationSystem [44], using the keys "Black poplar" and "POPULUS_NIGRA_RNASEQ_PANEL" for the fields "Crops" and "Germplasm list", respectively.

We described 14 of the 17 phenotypic traits in previous work [24]. Briefly, these traits can be divided into two categories, growth traits and biochemical traits which were all evaluated on up to 6 clonal replicates by genotype at each site after two years of growth in the second cycle. The first set is composed of the circumference of the tree at a 1-meter height measured in Savigliano at the end of 2009 (CIRC2009.Sav) and in Orléans at the end of 2011 (CIRC2011.Orl). The second set is composed, each time at both sites, of measures of ratios between the different components of the lignin, p-hydroxyphenyl (H), guaiacyl (G) and syringyl (S) (H.G.Orl, H.G.Sav, S.G.Orl and S.G.Sav), measures of the total lignin content (Lignin.Orl : measure of the lignin in Orléans, Lignin.Sav: measure of the lignin in Savigliano), measure of the total glucose (Glucose.Orl and Glucose.Sav), measure of ratio between 5 and 6 carbon sugars (C5.C6.Orl and C5.C6.Sav) and measure of the extractives (Extractives.Orl and Extractives.Sav). For each of these traits, we computed mean values per genotype previously adjusted for microenvironmental effects (block or spatial position in the field).

The 3 remaining traits were measured in 2015 on the trees harvested for the RNA sequencing experiment (2 replicates per genotype). They include the mean diameter of the stem section harvested for RNA sequencing (MeanDiameter), the date of bud flush of the tree in 2015 (Date3Doy) and the basic density of the wood (Infraden). Date of bud flush consisted of a prediction of the day of the year at which the apical bud of the tree was in stage 3 according to the scale defined in Dillen et al. [45]. Predictions were done with a lowess regression from discrete scores recorded at consecutive dates in the spring of 2015. Wood’s basic density was measured on a piece of wood from the stem section harvested for RNA sequencing following the Technical Association of Pulp and Paper Industry (TAPPI) standard test method T 258 "Basic density and moisture content of pulpwood".

### Transcriptome data generation

We sampled stem sections of approximately 80 cm long starting at 20 cm above the ground and up to 1 meter in June 2015. The bark was detached from the trunk in order to scratch young differentiating xylem and cambium tissues using a scalpel. The tissues were immediately immersed in liquid nitrogen and crudely ground before storage at -80 ^∘^C pending milling and RNA extraction. Prior to RNA extraction, the samples were finely milled with a swing mill (Retsch, Germany) and tungsten beads under cryogenic conditions with liquid nitrogen during 25 seconds (frequency 25 cps/sec). About 100 mg of milled tissue was used to isolate separately total RNA from xylem and cambium of each tree with RNeasy Plant kit (Qiagen, France), according to manufacturer’s recommendations. Treatment with DNase I (Qiagen, France) to ensure the elimination of genomic DNA was made during this purification step. RNA was eluted in RNAse-DNAse free water and quantified with a Nanodrop spectrophotometer. RNA from xylem and cambium of the same tree were pooled in an equimolar extract (250 ng/ *μ*L) before sending it to the sequencing platform.

RNA-seq experiment was carried out at the platform POPS (transcriptOmic Platform of Institute of Plant Sciences - Paris-Saclay) thanks to IG-CNS Illumina Hiseq2000. RNA-seq libraries were constructed using TruSeq_Stranded_mRNA_SamplePrep_Guide_15031047_D protocol (Illumina®, California, U.S.A.). The RNA-seq samples have been sequenced in single-end reads (SR) with an insert library size of 260 bp and a read length of 100 bases. Images from the instruments were processed using the manufacturer’s pipeline software to generate FASTQ sequence files. Ten samples by lane of Hiseq2000 using individually barcoded adapters gave approximately 20 millions of SR per sample. We mapped the reads on the *Populus trichocarpa* v3.0 transcriptome with bowtie2 v2.4.1 [46], and obtained the read counts for each of the 41,335 transcripts by homemade scripts (a median of 17 millions of reads were mapped per sample, with a minimum of 6 and a maximum of 42 million). *Populus trichocarpa* is considered the reference genome for the *Populus* genus with a high quality annotation, which is why we used it to map and quantify our data. In addition, the coding region is highly conserved between the two species and, as a result, 94% of our reads mapped on the *Populus trichocarpa* reference. Initially, we considered using the genotype means to reduce our data volume. However, differences between replicates were not normally distributed, because of variation in gene expression due to plasticity. We thus could not summarize our data with their mean, as it would have removed this information and finally we chose to keep replicates as separate data samples.

### Filtering the non-expressed genes, normalization and transformation to obtain a Gaussian distribution

We started cleaning our raw count data by removing the transcripts without at least 1 count in 10% of the individuals. From the original 41,335 genes, 7,106 were thus removed, leaving 34,229 genes. After this first filtration, we normalized the raw count data by Trimmed Mean of M-values (TMM, edgeR v3.26.4 [47]). As most features are not differentially expressed, this method takes into account the fact that the total number of reads can be strongly influenced by a low number of features. Then, we calculated the counts per millions (CPM [48]).

To make the CPM data fit a Gaussian distribution, we computed a *log*_2_(*n*+1) instead of a *log*_2_(*n*+0.5) typically used in a voom analysis [48], to avoid negative values, which are problematic for the rest of the analysis.

### Computing the BLUP, heritability, and *Q*_*ST*_ while correcting the co-variables

As the sampling ran along 2 weeks, we expected environmental variables to blur the signal. To understand how our data were impacted, we ran a PCA analysis to identify the impact of each cofactor (Suppl. Fig. 1). We identified the block and the sampling date and time as cofactors with a substantial impact.

A 12k bead chip [25] provided 7,896 SNPs in our population. A genomic relationship matrix between genotypes was computed with these SNPs with LDAK [49], and further split into between (mean population kinship, *K*_*b*_) and within-population relationship matrices (kinship kept only for the members of the same population, all the others are equal to 0, *K*_*w*_). These matrices were used in a mixed linear model to compute the additive genetic variances between and within populations for the expression of each gene:

1 $$  \mathbf{y} = \beta_{0} + \mathbf{Z_{b}} \mathbf{b} + \mathbf{Z_{w}} \mathbf{w} + \mathbf{\epsilon}  $$

Where, **y** is a gene expression vector across individual trees, *β*_0_ is a vector of fixed effects (overall mean or intercept); **b** and **w** are respectively random effects of populations and individuals within populations, which follow normal distributions, centered around 0, of variance $\sigma _{b}^{2} \mathbf {K_{b}}$ and $\sigma _{w}^{2} \mathbf {K_{w}}$. *σ*_*b*_ and *σ*_*w*_ are the between and within-population variance components and *K*_*b*_ and *K*_*w*_ are the between and within-population kinship matrices. *Z*_*b*_ and *Z*_*w*_ are known incidence matrices between and within populations, relating observations to random effects **b** and **w**. **ε** is the residual component of gene expression, following a normal distribution centered around 0, of variance $\sigma _{\epsilon }^{2} \mathbf {I}$, where *σ*_*ε*_ is the residual variance and **I** is an identity matrix.

We used the function "remlf90" from the R package breedR (v0.12.2) [27] to fit the model, with the Expectation-Maximization method followed by one round with Average-Information algorithm to compute the standard deviations. From the resulting between and within-population variance components, we computed the best linear unbiased predictors of between and within population random genetic effects ($\hat {\mathbf {b}}$ and $\hat {\mathbf {w}}$, respectively) and summed them up to obtain the total genetic value for each gene expression (*BLUP*). We also computed heritability (*h*^2^) and population differentiation estimates (*Q*_*ST*_) for each gene expression as follows:

2 $$  h^{2} = \frac{\sigma_{b}^{2} + \sigma_{w}^{2}}{\sigma_{b}^{2} + \sigma_{w}^{2} + \sigma_{\epsilon}^{2}}  $$

3 $$  Q_{ST} = \frac{\sigma_{b}^{2}}{\sigma_{b}^{2} + 2\sigma_{w}^{2}}  $$

Finally, we computed for each gene expression the coefficient of genetic variation (*CV*_*g*_) by dividing its total genetic variance ($\sigma _{b}^{2} + \sigma _{w}^{2}$) by its expression mean.

### Other population statistics

We further used a previously developed bioinformatics pipeline to call SNPs within our RNA sequences [50]. Briefly, this pipeline involves cleaning and quality control steps, mapping on the *P. trichocarpa* v3.0 reference genome, and SNP calling using the combination of four different callers. We ended up with a set of 874,923 SNPs having less than 50% of missing values per genotype. The missing values were further imputed with the software FImpute [51]. We validated our genotyping by RNA sequencing approach by comparing the genotype calls with genotyping previously obtained with an SNP chip on the same individuals [25]. Genotyping accuracy based on 3,841 common positions was very high, with a mean value of 0.96 and a median value of 0.99. The imputed set of SNP was then annotated using Annovar [52] in order to group the SNPs per gene model of *P. trichocarpa* reference genome. For each SNP, we computed the overall genetic diversity statistics with the hierfstat R package (v0.4.22) [53] and this statistic was then averaged by gene model in order to get information on the extent of diversity. We further computed *PCadaptscore* with the pcadapt R package (v4.3.3) [28] with 8 retained principal components. Here again, *PCadaptscores* were then summarized (averaged) by gene-model in order to get information about their potential involvement in adaptation. Based on the principal component analysis, pcadapt is more powerful to perform genome scans for selection in next-generation sequencing data than approaches based on *F*_*ST*_ outliers detection [28]. We found a positive correlation between *F*_*ST*_ and *PCadaptscore* (data not shown), but *PCadaptscore* highlighted differences between Core, random and peripheral gene sets (Fig. 3) while *F*_*ST*_ did not.

### Hierarchical clustering

We performed a weighted correlation network analysis with the R package WGCNA (v1.68) [5] on our full RNA-seq gene set. We followed the recommended approach, except that we first ranked our expression data, to work subsequently with Spearman’s non-parametric correlations and avoid problems due to linear modeling assumptions. We first chose the soft threshold with a power of 12, which is the recommended value for signed networks (and default value in WGCNA) (*R*^2^=0.81, connectivity: mean = 195.17, median = 9.23, max = 1403.96, Fig. 2a). Then, we used the automatic module detection (function "blockwiseModules") via dynamic tree cutting with a merging threshold of 0.25, a minimum module size of 30 and bidweight midcorrelations (Fig. 2b). All other options were left to default. This also computes module eigengenes. To sort the traits, we clustered their scaled values with the pvclust R packages (v2.2.0) [54], the Ward agglomerative method ("Ward.D2") on correlations (Fig. 2b, Fig. 2c, Suppl. Fig. 2). The clustering on euclidean distance results in the exact same hierarchical tree. Correlations between traits and gene expression or module eigengenes were computed as Spearman’s rank correlations (Fig. 2b, c).

### Machine learning

#### Boruta gene expression selection

In addition to the inconvenience of working with a large number of features (time and power consumption), most machine learning algorithms perform better when the number of predicting variables used is kept as low as the optimal set [55]. We thus performed an all relevant variable selection [56] with the Boruta function [29] from the eponym R package, with 4 *p*-value thresholds (1, 5, 10 and 20%), on the training subpart of the full gene expression set, for each phenotype independently. Then, features that were not rejected by the algorithm were pooled together, so that all the important genes were in the selected gene pool, one pool for each *p*-value threshold. The enrichment or depletion in core or peripheral genes in each of these pools was evaluated by Fisher’s exact test for count data ("fisher.test" function in the stats R package (v3.6.3) with default parameters).

#### Models

Both additive linear model (ridge regression) and interactive neural network models were computed by the R package h2o (v3.30.0.2) [57]. They both used the gene expression sets as predictors and one phenotypic trait at a time as a response. Datasets were split by the function "h2o.splitFrame" into 3 sets, a training set, a validation set and a test set, with the respective proportions of 60%, 20%, and 20%. We checked that the split preserves the distribution of samples within populations. The training set was used to train the models, the validation set was used to validate and improve the models, while the test set was used to compute and report prediction accuracies as *R*^2^ between observed and predicted values within this set and using the function "R2" of the R package compositions (v1.40.2) [58]. This set has never been used to improve the model and therefore represents a proxy of new data, avoiding the report of results from overfitted models. All the reported predictions scores were computed on this test set. These results are thus representing real-life predictions and are not subject to over-fitting.

For linear models, we used the function "h2o.glm" with default parameters, except 2-folds cross-validation and alpha set at zero to perform a ridge regression. The same splits and score reporting methods were used.

Neural networks have the reputation to be able to predict any problem, based on the Universal approximation theorem [59, 60]. However, this capacity comes at the cost of a very large number of neurons in one layer, or a reasonable number of neurons per layer in a high number of layers. Both settings lead to difficult interpretation when very many gene expressions are involved. In that sense, we chose to keep our models simple, with two layers of a reasonable number of neurons. This obviously comes at the price of lower prediction power. However, we believe that these topologies give us the power to model 2 levels of interactions between genes (1 level per layer). Furthermore, since both methods yielded comparable prediction *R*^2^ (median ridge regression *R*^2^=0.19, mean neural network *R*^2^=0.173), this complexity seemed appropriate. To find the best models for neural networks, we computed a random grid for each response. We tested the following four hyperparameters: (i) activation function ("Rectifier", "Tanh", "RectifierWithDropout" or "TanhWithDropout"); (ii) network structure; (iii) input layer dropout ratio (0 or 0.2) (iv) L1 and L2 regularization (comprised between 0 and 1×10^−4^, with steps of 5×10^−6^). Network structure corresponded to the number of neurons within each of the two hidden layers, which was based on the number of input genes (*h*). The first layer was composed of *h*, $\frac {2}{3}h$ or $\frac {1}{3}h$ neurons. The second layer had a number of nodes equal or lower to the first one and was also composed of *h*, $\frac {2}{3}h$ or $\frac {1}{3}h$ neurons. This represented a total of 6 different structures. We performed a random discrete strategy to find the best search criteria, computing a maximum of 100 models, with a stopping tolerance of 10^−3^ and 10 stopping rounds. Finally, "h2o.grid" parameters were the following: the algorithm was "deeplearning", with 10 epochs, 2 fold cross-validation, maximum duty cycle fraction for scoring 0.025, and constraint for a squared sum of incoming weights per unit 10. All other parameters were set to default values. The best model was selected from the lowest RMSE score within the validation set.

## Supplementary information

**Additional file 1** Suppl. Table 1. Module membership of each gene.

**Additional file 2** Suppl. Table 2. Distribution of core, peripheral and peripheral no grey genes across modules.

**Additional file 3** Suppl. Fig. 1. PCA score plots on gene expression data. Each plot represents the distribution of the individuals on the 2 first axes of the PCA (representing 17,7% of the variation), colored by class of various experimental factors (Xylem and cambium scraper, extractor and extraction method, population, sequencing column, line and plate, the growth rate at harvest, sampling date, time, temperature, solar radiation, humidity and wind speed). Cofactors related to weather are presented in the 6 lower plots.

**Additional file 4** Suppl. Fig. 2. Traits hierarchical ascendant clustering dendrogram. Clustering was performed from the correlations between traits with Ward method ("Ward.D2") by the R package pvclust. Approximately Unbiased (au, in red) and Bootstrap Probability (bp, in green) *p*-values indicated the degree of belief associated with clusters. Highly supported modules are framed by a red square, grouping (a) the mean sample diameter with the two circumference traits, (b) the S/G ratios with glucose composition, (c) the two C5/C6 together, and (d) the H/G ratios.

**Additional file 5** Suppl. Fig. 3. Relationship between Spearman’s correlations between module-trait (y-axis) and gene significance-kME (x-axis).

**Additional file 6** Suppl. Fig. 4. Histograms of the centrality scores without (top) or with (bottom) the grey group. Core, peripheral and peripheral without grey sets are represented respectively by the blue, dark orange and orange bars. Random sets are distributed across the histogram and do not appear on this figure. Distribution of genes clustered in the grey module is represented by the grey bars, white bars are for other genes.

**Additional file 7** Suppl. Fig. 5. Histograms of the centrality scores for the genes selected by Boruta at different *p*-values thresholds. Repartition of selected genes within the following gene sets is hilighted, with core in blue, peripheral NG in orange, peripheral in brown and other (NA) in black. Four *p*-value thhresolds for Boruta selections were considered: 0.01, 0.05, 0.1 and 0.2.

**Additional file 8** Suppl. Fig. 6. Proportion of linear model (LM, top row) and neural network (NN, bottom row) predictions with a *R*^2^ above (left column) or below (right column) the 95% confidence interval computed from the predictions with the random sets of genes for each gene set (there is no neural network model computed for the Complete BLUP full set).

**Additional file 9** Suppl. Fig. 7. Difference of prediction scores between algorithms (top) and sets (bottom). On the top panel, the difference between LM and NN prediction scores for the core (in blue), random (in grey), peripheral (in brown), peripheral (in orange) and Boruta gene sets (in green). On the bottom panel, the LM differences are in red and the NN differences in turquoise and the color filling the bar represents the difference between core and peripheral genes in brown, core and peripheral NG in orange and between the random sets in grey. For the random pairs, error bars represent the first and third quartiles of the differences between pairs of randomized sets and the bar corresponds to the median.

**Additional file 10** Suppl. Fig. 8. Predictions scores on test sets for increasing numbers of the peripheral genes. Violin and boxplots of prediction *R*^2^ for the LM Ridge algorithm and for increasing sizes of the peripheral genes set (in brown) and the peripheral NG genes set (in orange), used for the predictions (in percent of the full set).

## Abbreviations

CPM: Counts per million
*CVg*: Genetic variation coefficient
DNA: Deoxyribonucleic acid
eQTL: Expression QTL
*F*_*ST*_: Fixation index
GWAS: Genome-wide association study
*h*^2^: Heritability
*Ht*: Overall genetic diversity statistic
kME: A measure of centrality in a module
LM: Linear model
NN: Neural networks model
ORL: Orléans common garden experiment
PCA: Principal component analysis
*PCadapt**score*: The score from analysis with PCadapt R package
*Q*_*ST*_: Coefficient of quantitative genetic differentiation
QTL: Quantitative trait loci
RNA: Ribonucleic acid
RNA-seq: RNA sequencing
SAV: Savigliano common garden experiment
SNP: Single nucleotide polymorphism
SR: Single-end read
TMM: Trimmed mean of m-values
WGCNA: Weighted gene correlation network analysis
