# Supplementary figures and images for: Gene expression predictions and networks in natural populations supports the omnigenic theory
Source: BMC Genomics. 2020 Jun 22;21:416. doi: 10.1186/s12864-020-06809-2 (PMC7310122; doi:10.1186/s12864-020-06809-2)

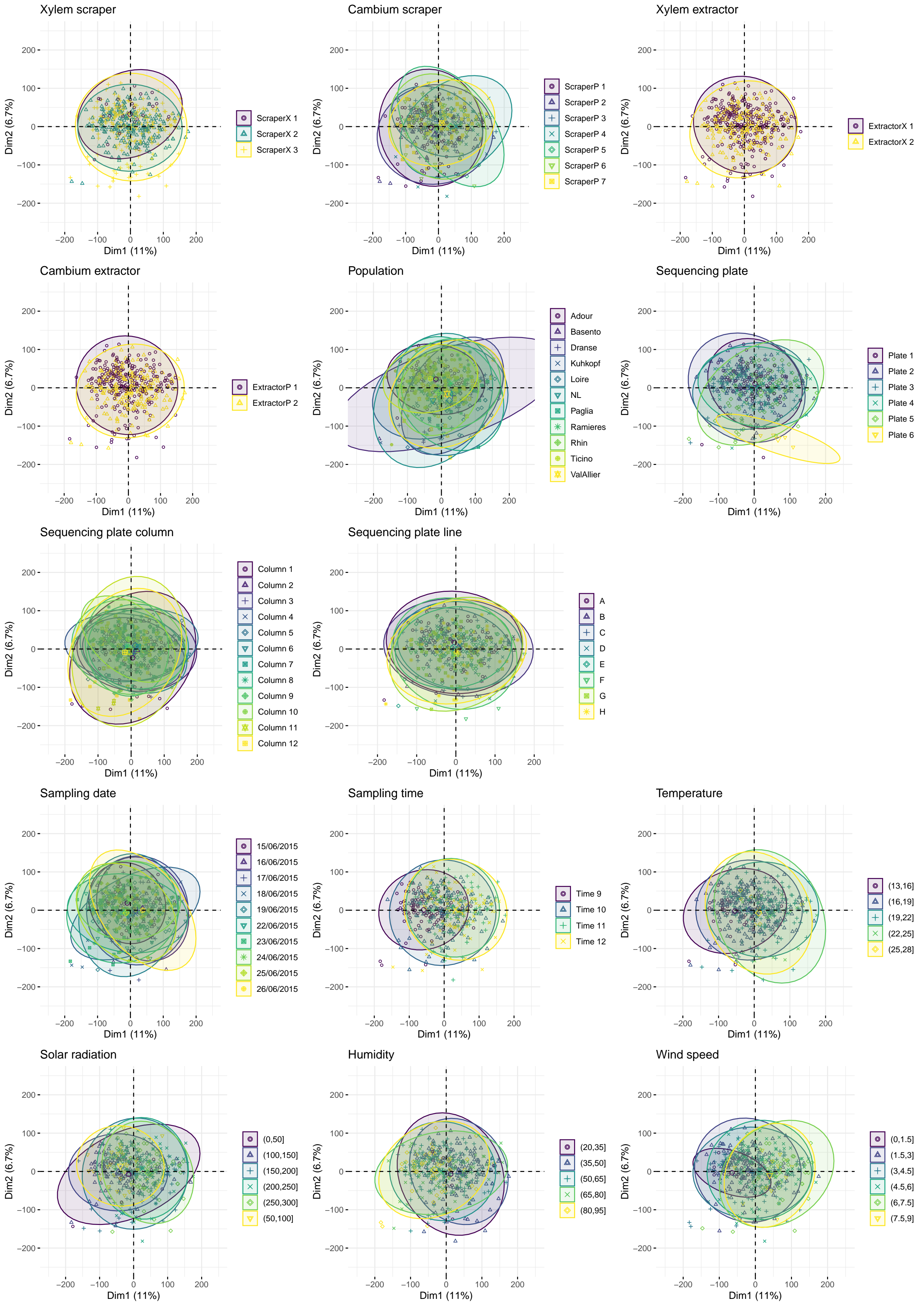

Supplement: Supplementary file 3 — Additional file 3 Suppl. Fig. 1. PCA score plots on gene expression data. Each plot represents the distribution of the individuals on the 2 first axes of the PCA (representing 17,7% of the variation), colored by class of various experimental factors (Xylem and cambium scraper, extractor and extraction method, population, sequencing column, line and plate, the growth rate at harvest, sampling date, time, temperature, solar radiation, humidity and wind speed). Cofactors related to weather are presented in the 6 lower plots. [file 12864_2020_6809_MOESM3_ESM.pdf]

# Cluster dendrogram with p-values (%)

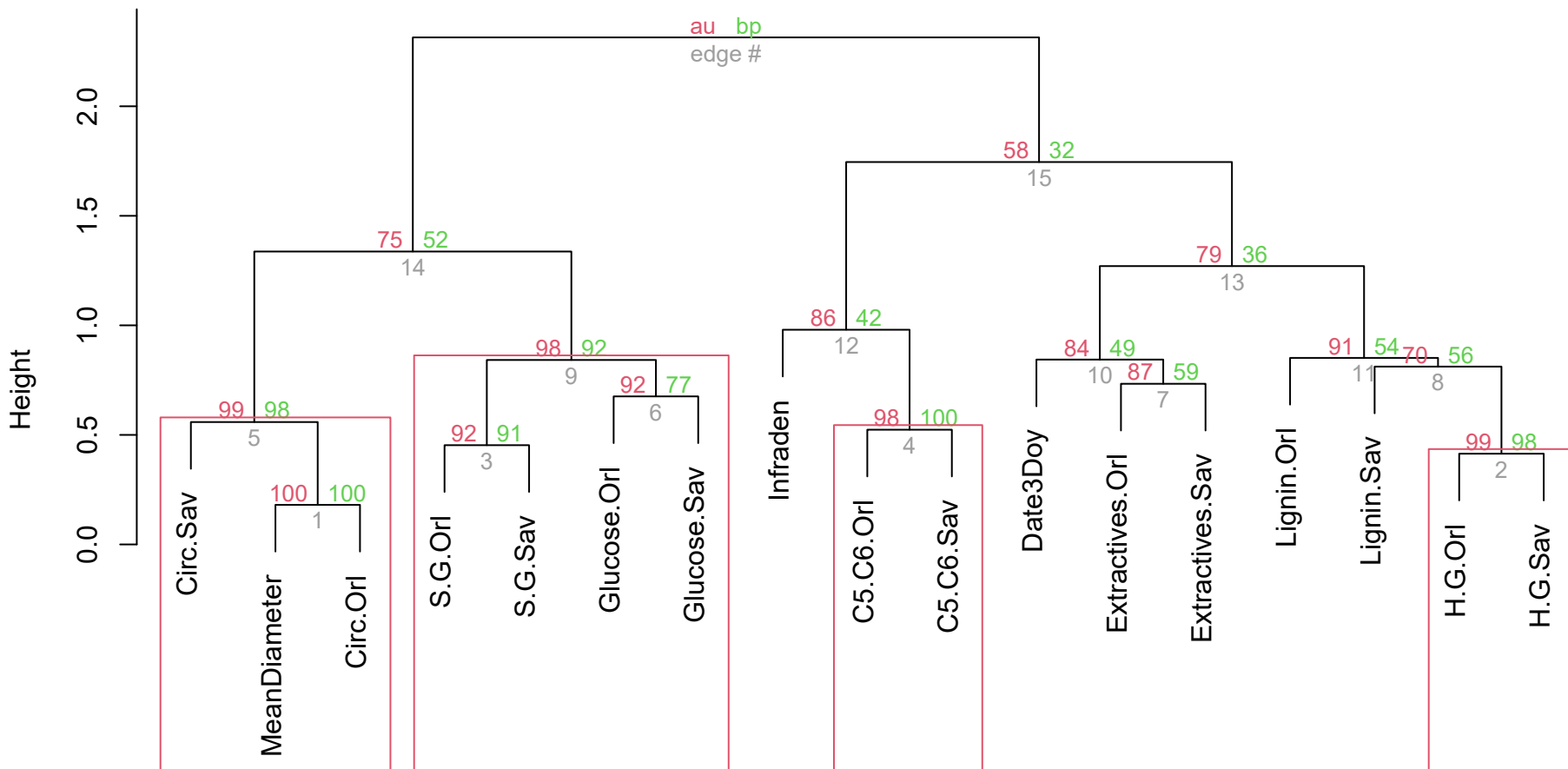

Supplement: Supplementary file 4 — Additional file 4 Suppl. Fig. 2. Traits hierarchical ascendant clustering dendrogram. Clustering was performed from the correlations between traits with Ward method ("Ward.D2") by the R package pvclust. Approximately Unbiased (au, in red) and Bootstrap Probability (bp, in green) p-values indicated the degree of belief associated with clusters. Highly supported modules are framed by a red square, grouping (a) the mean sample diameter with the two circumference traits, (b) the S/G ratios with glucose composition, (c) the two C5/C6 together, and (d) the H/G ratios. [file 12864_2020_6809_MOESM4_ESM.pdf]

Correlation between eigengene and trait

$R = 0.94$  ,  $p < 2.2e-16$

0.5

0.0

-0.5

-0.5

0.0

0.5

1.0

Correlation between gene significance and kME

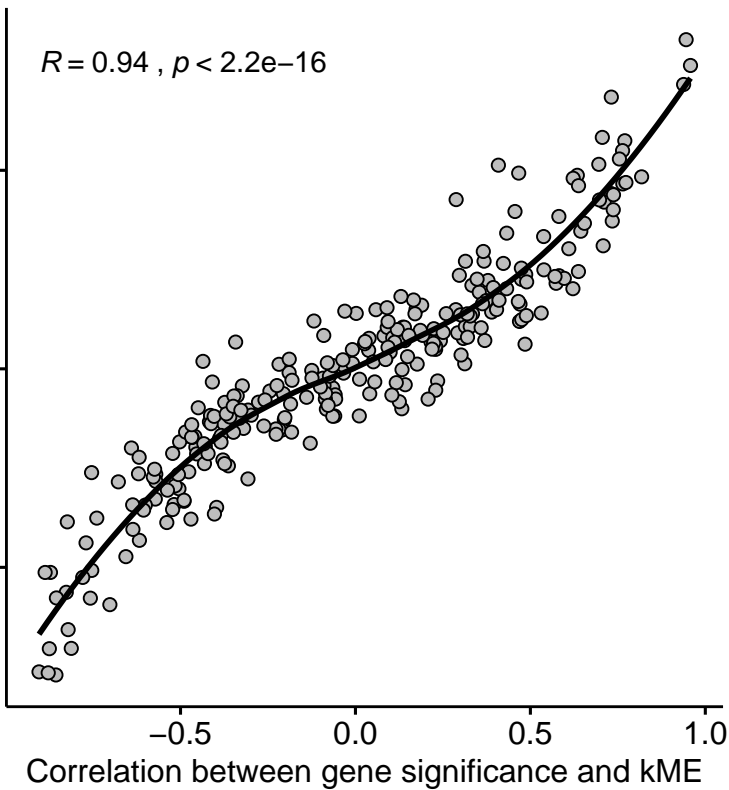

Supplement: Supplementary file 5 — Additional file 5 Suppl. Fig. 3. Relationship between Spearman’s correlations between module-trait (y-axis) and gene significance-kME (x-axis). [file 12864_2020_6809_MOESM5_ESM.pdf]

**Without the group grey**

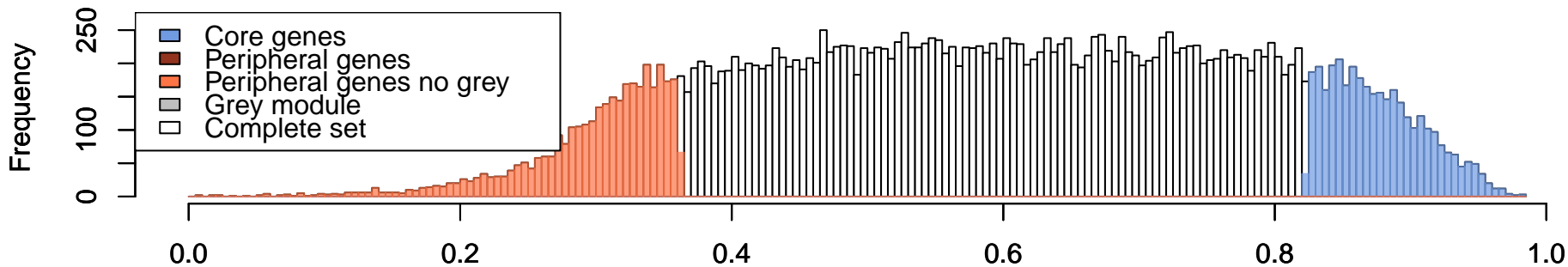

**With the group grey**

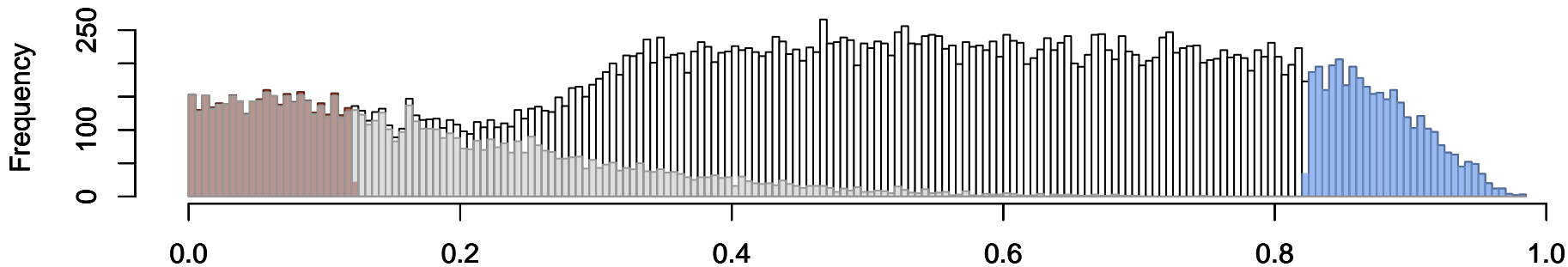

Supplement: Supplementary file 6 — Additional file 6 Suppl. Fig. 4. Histograms of the centrality scores without (top) or with (bottom) the grey group. Core, peripheral and peripheral without grey sets are represented respectively by the blue, dark orange and orange bars. Random sets are distributed across the histogram and do not appear on this figure. Distribution of genes clustered in the grey module is represented by the grey bars, white bars are for other genes. [file 12864_2020_6809_MOESM6_ESM.pdf]

set core peripheral NG peripheral NA

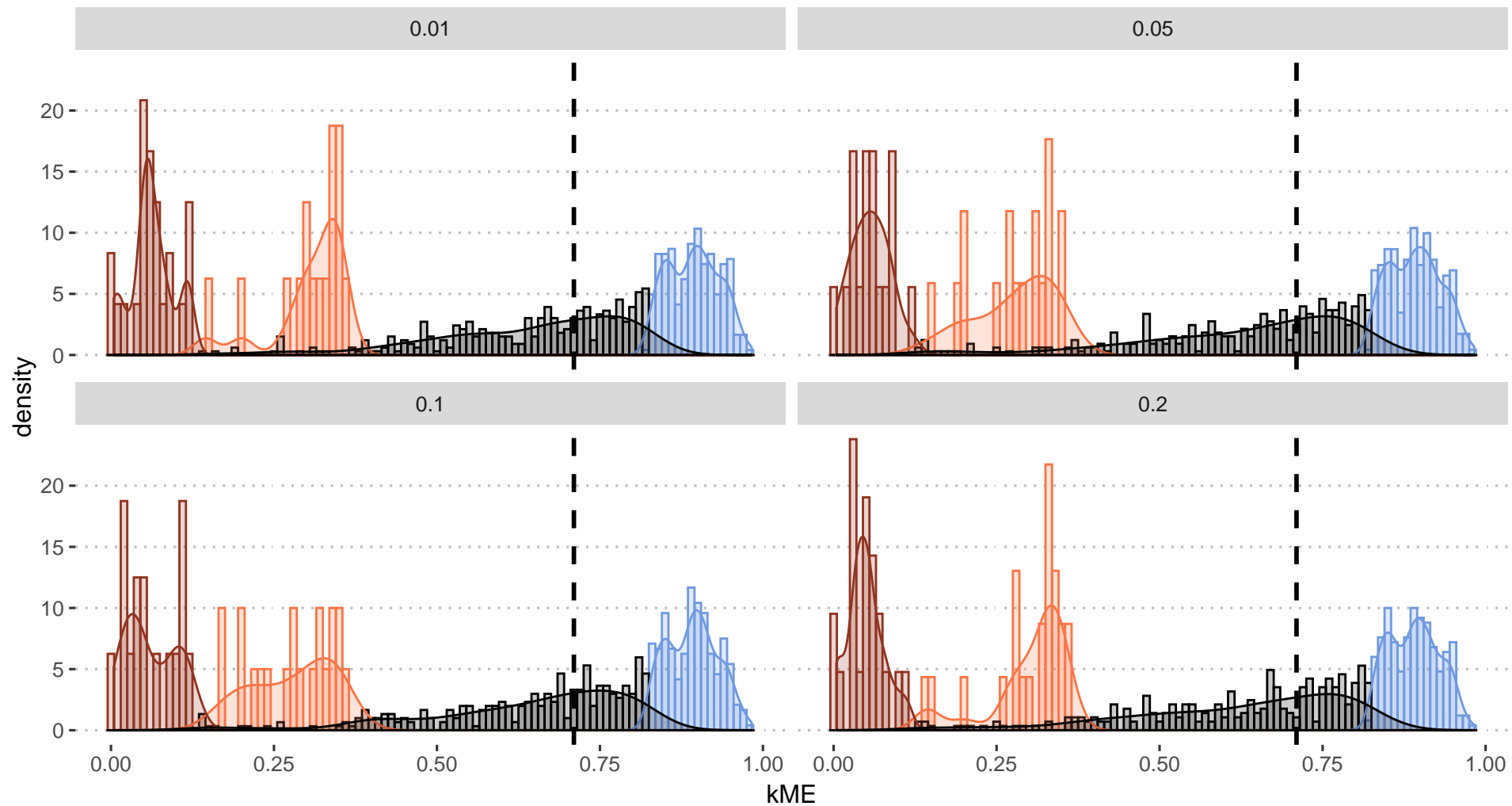

Supplement: Supplementary file 7 — Additional file 7 Suppl. Fig. 5. Histograms of the centrality scores for the genes selected by Boruta at different p-values thresholds. Repartition of selected genes within the following gene sets is hilighted, with core in blue, peripheral NG in orange, peripheral in brown and other (NA) in black. Four p-value thhresolds for Boruta selections were considered: 0.01, 0.05, 0.1 and 0.2. [file 12864_2020_6809_MOESM7_ESM.pdf]

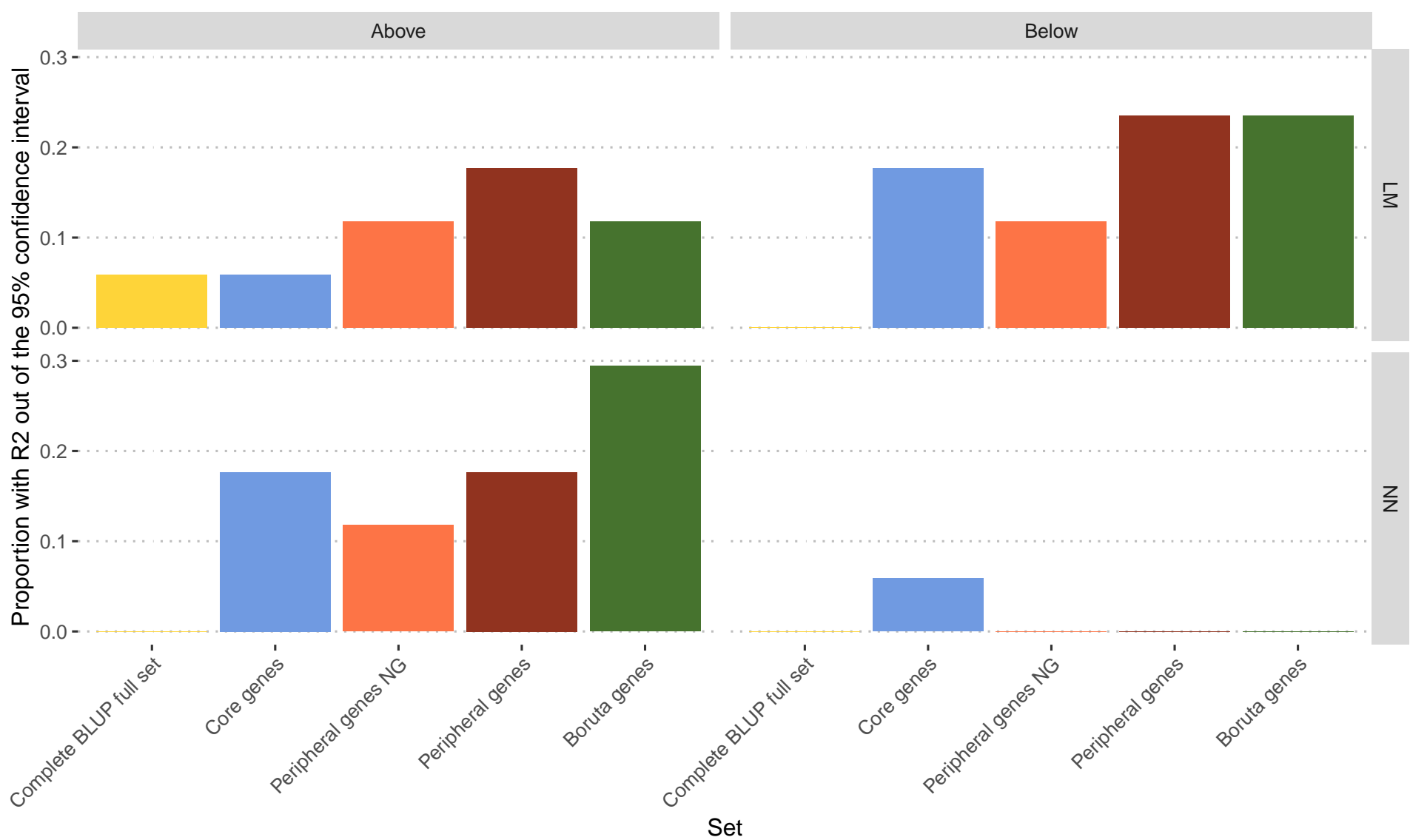

Supplement: Supplementary file 8 — Additional file 8 Suppl. Fig. 6. Proportion of linear model (LM, top row) and neural network (NN, bottom row) predictions with a R2 above (left column) or below (right column) the 95% confidence interval computed from the predictions with the random sets of genes for each gene set (there is no neural network model computed for the Complete BLUP full set). [file 12864_2020_6809_MOESM8_ESM.pdf]

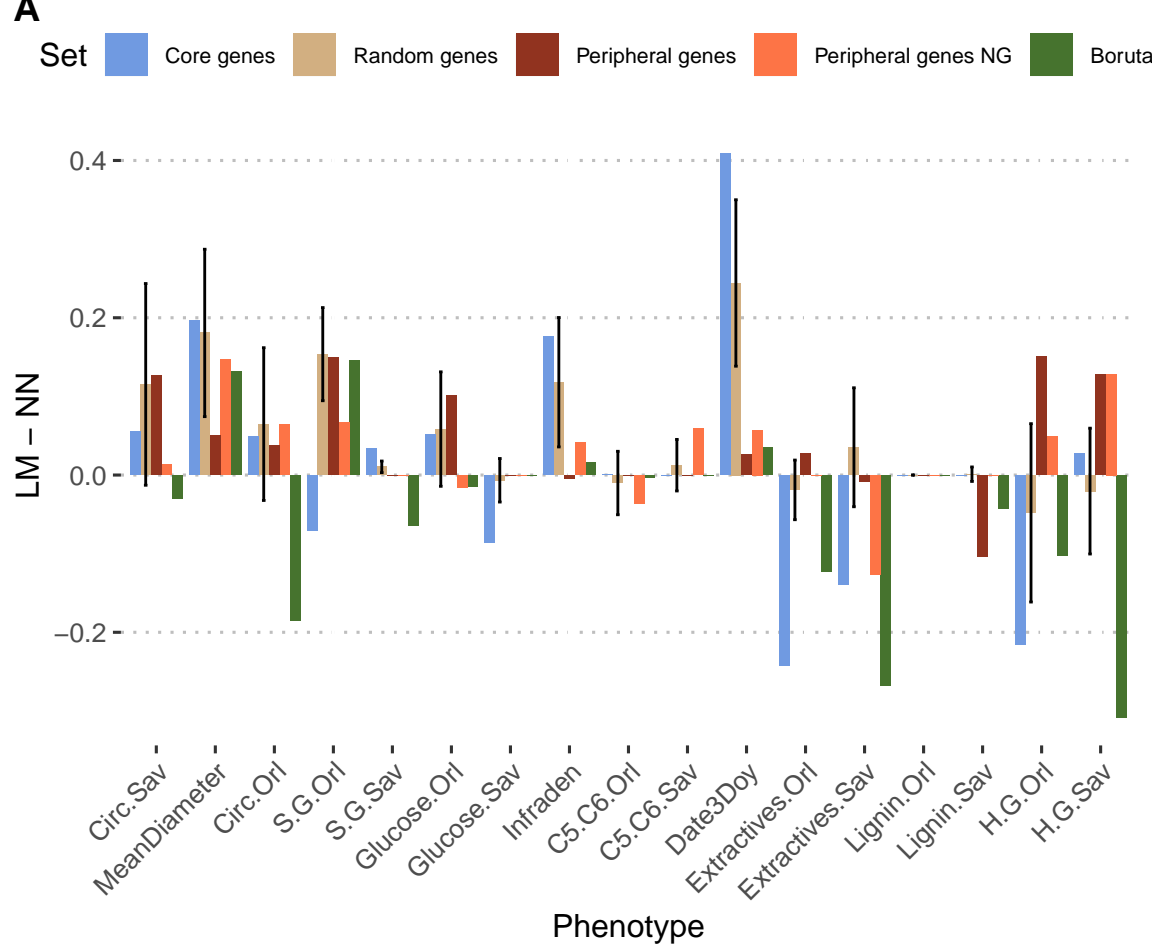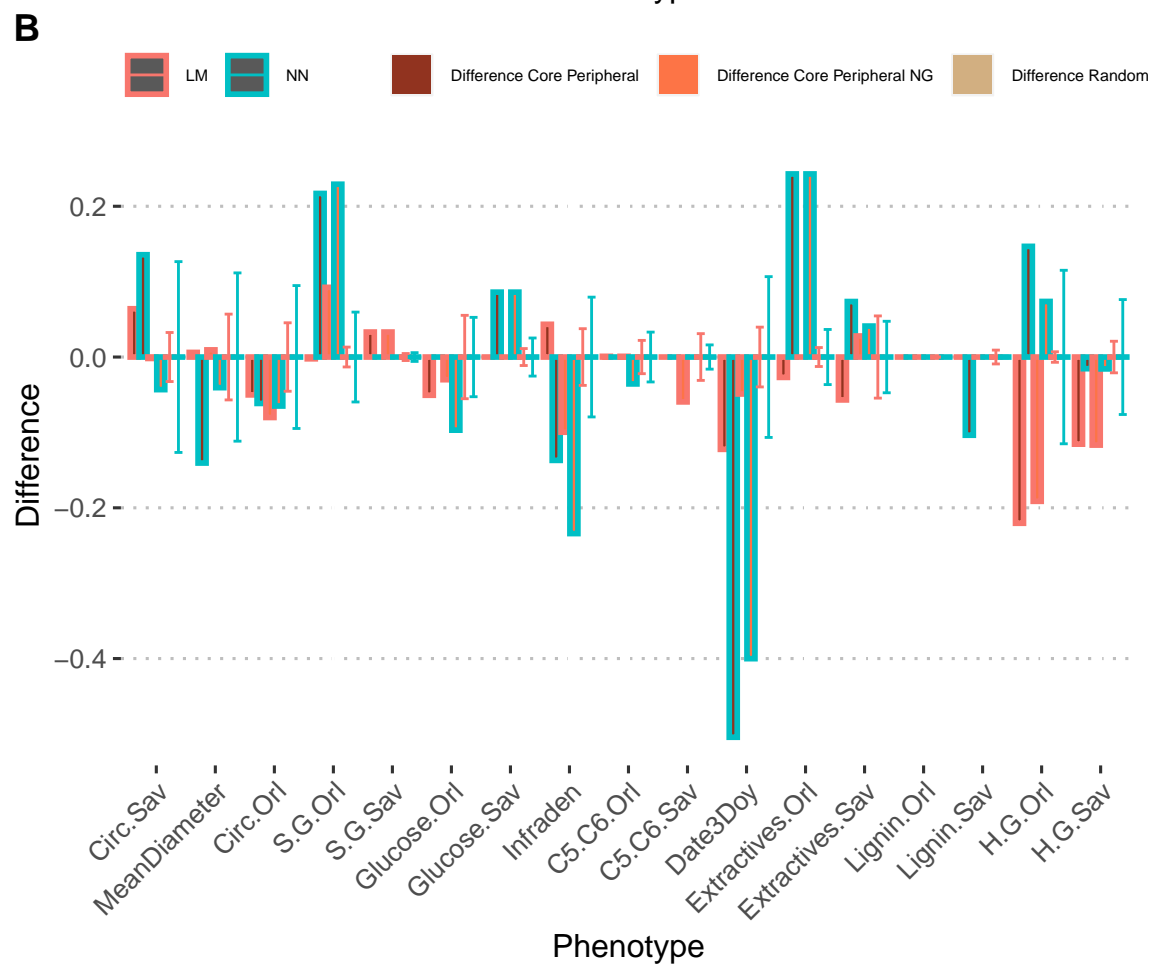

Supplement: Supplementary file 9 — Additional file 9 Suppl. Fig. 7. Difference of prediction scores between algorithms (top) and sets (bottom). On the top panel, the difference between LM and NN prediction scores for the core (in blue), random (in grey), peripheral (in brown), peripheral (in orange) and Boruta gene sets (in green). On the bottom panel, the LM differences are in red and the NN differences in turquoise and the color filling the bar represents the difference between core and peripheral genes in brown, core and peripheral NG in orange and between the random sets in grey. For the random pairs, error bars represent the first and third quartiles of the differences between pairs of randomized sets and the bar corresponds to the median. [file 12864_2020_6809_MOESM9_ESM.pdf]

set 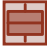 Peripheral genes 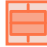 Peripheral genes NG

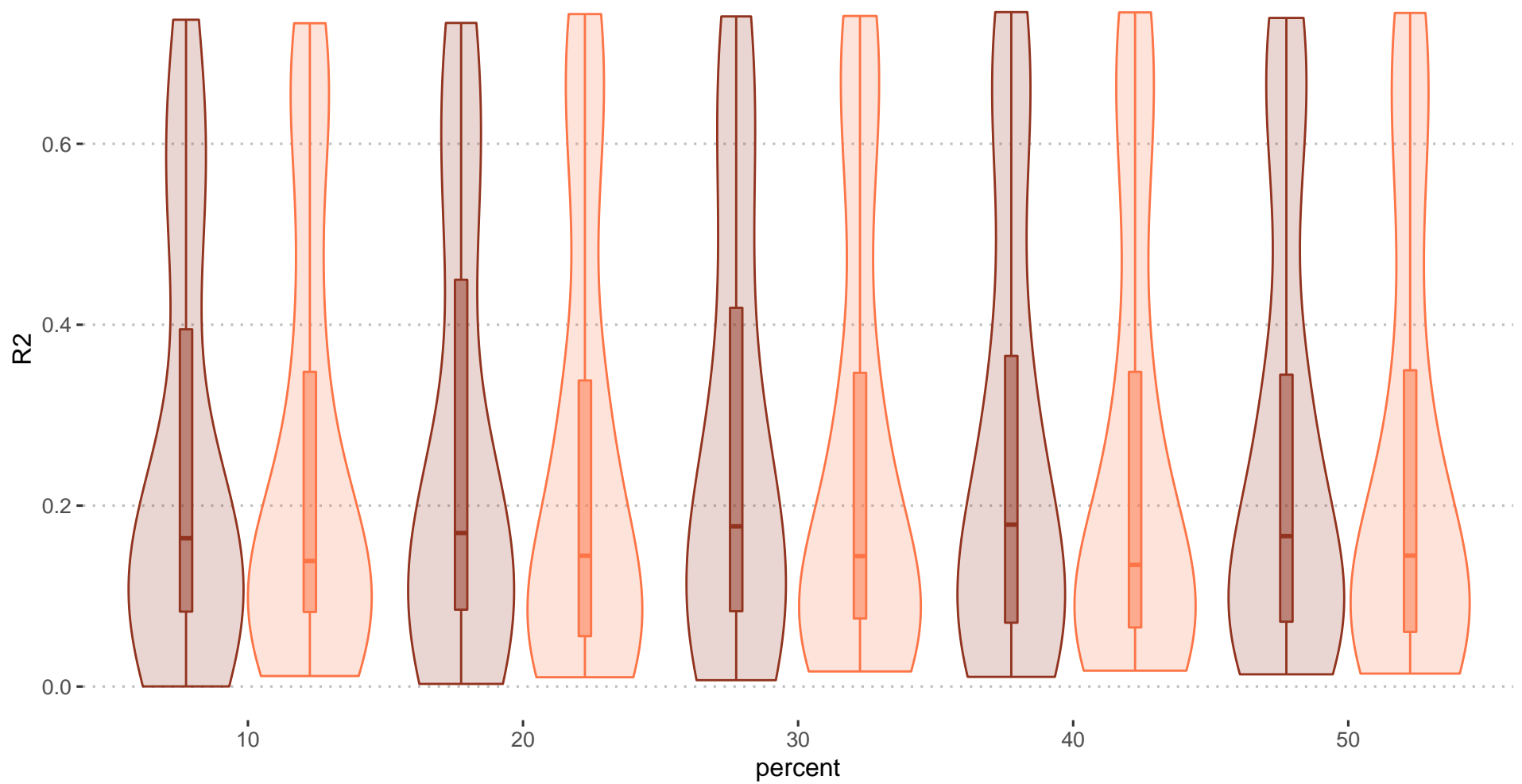

Supplement: Supplementary file 10 — Additional file 10 Suppl. Fig. 8. Predictions scores on test sets for increasing numbers of the peripheral genes. Violin and boxplots of prediction R2 for the LM Ridge algorithm and for increasing sizes of the peripheral genes set (in brown) and the peripheral NG genes set (in orange), used for the predictions (in percent of the full set). [file 12864_2020_6809_MOESM10_ESM.pdf]
